# HSP25 and HSP25-P-Ser15 Prompt Innate Neuroprotection in Lobe X of the Cerebellum

**DOI:** 10.3390/ijms27031145

**Published:** 2026-01-23

**Authors:** Carlos Hernández-Pérez, Laura Pérez-Revuelta, Pablo G. Téllez de Meneses, Valeria L. Cabedo, José Ramón Alonso, David Díaz, Eduardo Weruaga

**Affiliations:** 1 Laboratory of Neuronal Plasticity and Neurorepair, Institute for Neuroscience of Castilla y León (INCyL), Universidad de Salamanca, 37007 Salamanca, Spain; carlosh@usal.es (C.H.-P.); pabgonses@usal.es (P.G.T.d.M.); cabedonavarro.valeria@usal.es (V.L.C.); jralonso@usal.es (J.R.A.); 2 Institute of Biomedical Research of Salamanca (IBSAL), 37007 Salamanca, Spain; 3 Cologne Excellence Cluster on Cellular Stress Responses in Aging-Associated Diseases (CECAD), University of Cologne, 50923 Cologne, Germany; laura.perez-revuelta@uk-koeln.de

**Keywords:** cerebellar lobes, cerebellum, CONDCA, HSP, Purkinje Cell Degeneration, neuroresistance

## Abstract

The cerebellar cortex presents a repetitive structure, but the main projecting neurons of this tissue, the Purkinje cells, are not identical and behave differently to various types of injury. Common patterns of neurodegeneration exist, where certain Purkinje cells die earlier than others. By contrast, lobe X of the cerebellum is a particularly resistant structure, independently of the cerebellar disease or damage. However, the mechanisms underlying the survival capability of these especially resistant Purkinje cells are still unknown. In this work, we have used the Purkinje Cell Degeneration (PCD) mouse, a model of severe cerebellar degeneration that also reproduces the human disease called childhood-onset neurodegeneration with cerebellar atrophy, to study Purkinje cell resistance. After an exhaustive immunochemical analysis of the different subpopulations of Purkinje cells, the Heat Shock Protein 25 (HSP25) and its phosphorylated version HSP25-P-Ser15 were found to be especially induced in lobe X of PCD mice. As this protein has neuroprotective properties, it may be responsible for resistance against cerebellar neurodegeneration. Taking into account the constant resistance of lobe X, the use of HSP25 may lead to new possibilities for achieving natural protection both in cerebellum and in other brain structures, or even for developing future neuroprotective therapies.

## 1. Introduction

The cerebellar cortex presents structural patterns that have been conserved throughout evolution [1]. In vertebrates, it has a common stratification, comprising the molecular, Purkinje cell, and granule cell layers, with the Purkinje neurons being the projecting cells specific in this cortex [2]. However, beyond this repetitive structure, additional subdivisions can be distinguished using certain immunohistochemical markers such as Zebrin II. It is known that this polypeptide is selectively expressed in the cerebellum, from null to full expression, revealing a series of parasagittal bands that define four different regions in the cerebellar cortex: anterior (lobes I–V), central (lobes VI–VII), posterior (lobes VIII–IX), and nodular (lobe X) [3].

Despite its uniformity, the cerebellar cortex has different levels of sensitivity to damage depending on the region. The most delicate part generally coincides with the anterior lobes, whereas lobe X is intriguingly the most resistant region of the cerebellum [4]. Such resistance is common in many models of cerebellar degeneration, even those suffering different types of neural damage. One of these models, the Purkinje Cell Degeneration (PCD) mutant mouse, perfectly reflects this form of resistance [5] and is used as a model for a neurodegenerative disease affecting children called childhood-onset neurodegeneration with cerebellar atrophy (CONDCA) [6,7,8]. Both PCD mice and humans with CONDCA suffer dramatic shrinkage of the cerebellum due to the absence of cytosolic carboxypeptidase 1, which provokes the destabilization of the cytoskeleton and consequently Purkinje cell death [9]. The cerebellar neuronal degeneration found in this model is particularly aggressive, where at post-natal day 15 (P15), a short period of subcellular changes called the pre-neurodegenerative stage begins. Then, at P18, the neurodegenerative stage starts [10] followed by the almost complete death of all Purkinje cells at P30, except in lobe X, where neurodegeneration starts later and is milder than in the other lobes [4,5,11]. In PCD mice, Purkinje cell death occurs via apoptotic-like mechanisms, as evidenced by internucleosomal DNA fragmentation and markers of a non-conventional apoptotic process [11]. Indeed, some aberrant Purkinje cells do remain here at 9 months of age. Then, it is astonishing how lobe X resists cell death even under the aggressive neurodegeneration observed in PCD mice. Although we have recently detected certain specific mechanisms of neuroresistance in lobe X of PCD mice [12], this phenomenon has been also observed in several other models of cerebellar damage [4]. However, the subjacent factors to this generalized resistance are not fully understood. Initially, Zebrin II was singled out as a possible cause for the resistance of lobe X owing to its ubiquitous expression in this region [3]. However, this theory was discarded because other models of cerebellar neurodegeneration, such as the Nervous mouse, where Purkinje cells labeled positive for Zebrin II, are precisely the most vulnerable [13]. Therefore, Zebrin II appears to merely distinguish different cell populations or cell types with varying degrees of sensitivity depending on the stimulus received.

Alternatively, there is a family of proteins that may be more suitable candidates than Zebrin II for promoting the resistance found in lobe X; namely, heat shock proteins (HSPs) that have various characteristics and functions. First, HSP production can be induced by different forms of stress, such as ischemia, viruses, toxic compounds, etc., and act as chaperones and prevent the precipitation of other proteins [14]. HSPs also behave as chaperones by helping new proteins to fold when they are constitutively expressed [14,15]. Additionally, HSPs have other functions such as helping in the transport of other proteins [16], influencing the immune response [17], and inhibiting apoptosis [18].

HSPs weighing less than 34 kDa are called small HSPs (sHSPs) and there is a wide variety of this type of protein. However, the 25 kDa Heat Shock Protein (HSP25), or its homolog in mammals, HSP27, is of particular interest due to its neuroprotective effects in murine models. This protein is constitutively expressed in the cerebellar cortex of mice and Purkinje cells immunoreactive to HSP25 are grouped in symmetrical parasagittal bands in the vermis and the floccular and parafloccular zones [15]. An example of the neuroprotective role of HSP25 can be found in the Lurcher mouse, a model of cerebellar degeneration carrying a mutation in a δ2 glutamate receptor gene that results in Purkinje cell death. In this model, neurons of lobe X are more resistant and the constitutive expression of HSP25 seems to be one of the underlying factors of this condition [19]. Additionally, another model of cerebellar degeneration, the NPC1 mouse, suffers neuronal degeneration that progresses throughout the cerebellar cortex, although lobe X is the least affected of all four lobes [20]. Furthermore, the presence of HSP25 in the lobe X of NPC1 mice has been shown, and the inhibition or the induction of this protein increased or decreased, respectively, disease-associated symptoms [21].

In addition, the homolog of HSP25, HSP27, which acts as a chaperone, is involved in actin stability and protein folding, counteracts oxidative damage, and prevents apoptosis [22]. HSP27 in non-murine models acts as a direct inhibitor of apoptosis at different levels, where it blocks cytochrome c [23] and caspase-3 [24], and inhibits BAX activation [25] and DAXX signaling [26], all important steps in the pathway of apoptosis signaling. These processes are not carried out by native forms of HSP25/27, but require them to be phosphorylated, which can occur at different serine residues (HSP-P-Ser): Ser-15, Ser-78, Ser-82, or Ser-86, depending on the homolog [27]. However, not all phosphorylation can confer anti-apoptotic activity. Only HSP25/27-P-Ser15 and HSP25/27-P-Ser86 have this activity, and protein kinase C-δ (PKC-δ) can phosphorylate them in the nervous system [28]. Thus, in the NPC1 model, HSP25-P-Ser15 and PKC-δ are co-expressed in the posterior and nodular lobes [21].

In sum, HSP25 and its homologs are widely expressed in lobe X of several animal models, conferring protective effects to this region against moderate neuronal loss. However, for PCD mice, the model for the human disease CONDCA, characterized by severely aggressive neurodegeneration and resistance to cell death in lobe X, information on HSP25 is scarce. Therefore, determining whether this protein has the same role in this highly sensitive model is of particular interest for situating HSP25 as a possible common denominator for the resistance inherent to lobe X.

## 2. Results

### 2.1. PCD Mice Show Higher Rates of HSP25 Expression

**Figure 1:**
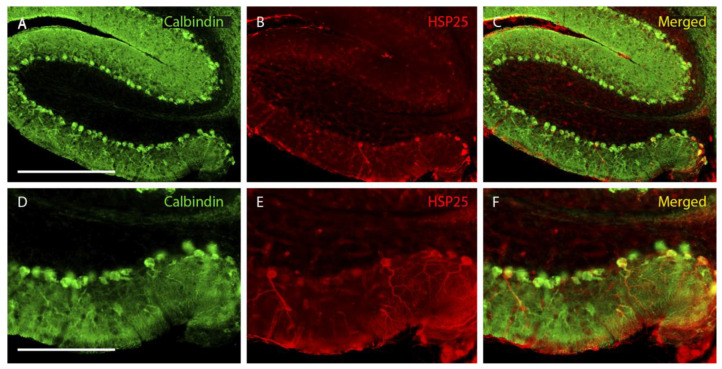
**Wild-type lobe X at P25.** (**A**–**C**), images showing wild-type Purkinje cells labeled with calbindin (green) and HSP25 (red). (**D**–**F**), magnified images of the ventral part of (**A**–**C**). Note that the HSP25 labeling in wild-type mice is exclusively located in the ventral-most area of lobe X. *n* = 5 for this experiment. Scale bars are 500 µm for (**A**–**C**) and 200 µm for (**D**–**F**).

**Figure 2:**
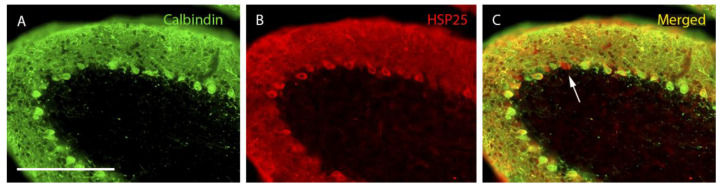
**PCD lobe X at P30.** Purkinje cells labeled with calbindin (**A**, green), HSP25 (**B**, red), and both markers (**C**, merged). Immunoreactivity for HSP25 is distributed homogeneously throughout the entire extension of the Purkinje cell layer of lobe X in PCD mice. In （**C**）, a Purkinje cell expressing HSP25 but not calbindin can be distinguished (arrow). *n* = 7 for this experiment. Scale bar 400 µm.

In lobe X of the wild-type animals, not all the Purkinje cells expressing calbindin were HSP25 positive. In contrast, all cells expressing HSP25 also expressed calbindin (Figure 1). Moreover, it was striking that HSP25-positive Purkinje cells in the lobe X of wild-type animals were mainly located in its ventral-most zone (Figure 1). Regarding PCD mice, its cerebellar cortex degenerated rapidly, losing a large number of Purkinje cells to the point that at P30, only small populations or isolated cells could be observed (Supp. Figure S1). However, the lobe X, at this age, appeared to be intact, as previously described [4] (Supp. Figure S1). Conversely, a higher expression of HSP25 was observed in PCD animals than in wild-type mice, since the Purkinje cells positive for HSP25 appeared throughout the entire extension of lobe X (Figure 2). In addition, some cells positive for HSP25 but without staining for calbindin were observed at the oldest ages analyzed (Figure 2).

**Figure 3:**
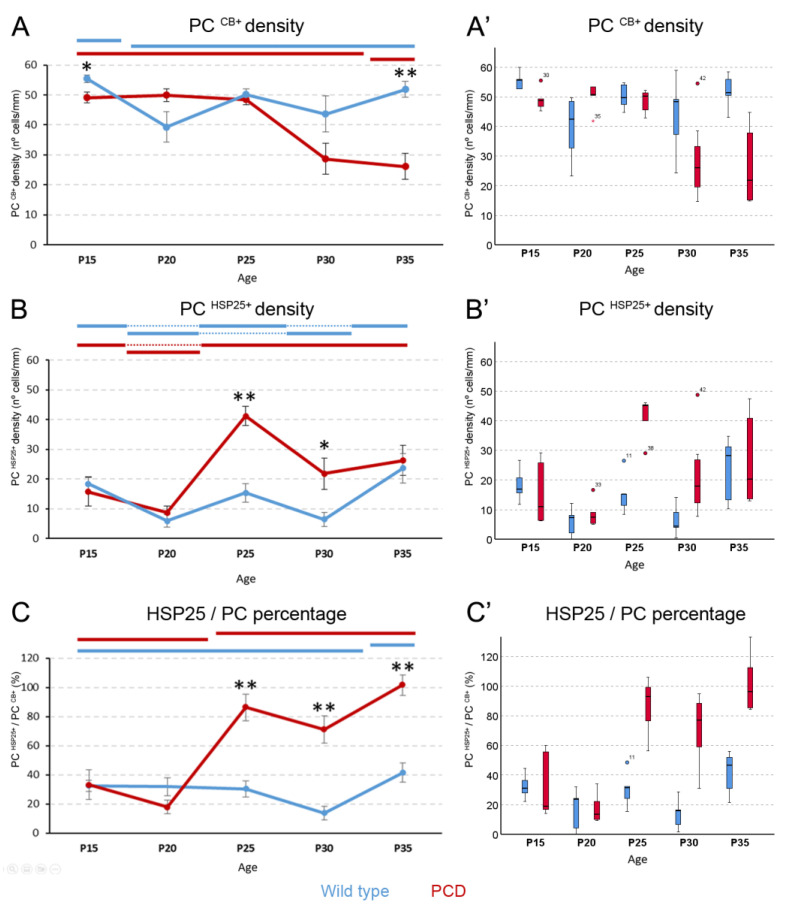
**Measures of HSP25 in Purkinje cells.** (**A**), chart showing Purkinje cell density of lobe X over time. Wild-type animals show the highest values for Purkinje cell density at P15, which then decreases and remains constant during the rest of the ages. The PCD lobe X has the same Purkinje cell density as the controls at P20, P25, and P30, but at P35, neurodegeneration becomes evident and cell density decreases. (**B**), chart depicting HSP25-positive cell density in lobe X over time. Both genotypes present fluctuations, especially the wild-type mice, but increased HSP25 expression was seen in PCD lobe X at P25 and P30 compared to wild-type mice. (**C**), chart displaying the ratio (expressed in %) of HSP25-positive neurons in the total number of Purkinje cells. Both genotypes increase this ratio over time, with PCD doing so earlier than wild-type mice; moreover, PCD animals have higher values than wild-type animals from P25 onward. In (**A**–**C**), data are expressed as the mean ± standard error of the mean for easing the comprehension of results. When the Kruskal–Wallis test showed significant differences among ages within each experimental group, a *post hoc* test was applied to define statistically similar groups (subset of values). To identify these subsets and avoid blurring of data and symbols, upper horizontal lines were drawn in each graph for each experimental group (blue, wild type; red, PCD). Note that lines at the same height imply comparable values. (**A’**–**C’**), box plot representations of the same data in (**A**–**C**), for a better visualization of their distribution, as they were analyzed with non-parametric tests; note that no marks of signification were represented here to avoid blurring the data. PC, Purkinje cell. *n* = 5 for each genotype and age, except for PCD at P30 (*n* = 7) and PCD at P35 (*n* = 8). * *p* < 0.05 and ** *p* < 0.01 for comparisons between wild-type and PCD mice at the corresponding age.

After quantifying calbindin-expressing cells, surprisingly, differences among ages were detected in the wild-type mice, a phenomenon that has not been described in previous studies. The wild-type animals presented a reduction in Purkinje cell density after P15; however, this parameter remained onwards constant (*p*-value for age comparisons in wild-type mice or pWT = 0.035; Figure 3A). By contrast, in PCD mice, variations in Purkinje cell density behaved differently over time: the neuronal density remained stable from P15 to P30 but decreased at P35 (*p*-value for age comparisons in PCD mice or pPCD = 0.002 Figure 3A). When comparing calbindin expression between both genotypes, we first observed that at P15, the wild-type lobe X had a greater Purkinje cell density than in the PCD counterpart (*p* = 0.032; Figure 3A). At P20, P25, and P30, the density of these neurons was the same in both genotypes (*p* > 0.05 in all cases; Figure 3A). However, later on, neurodegeneration in the PCD mouse became more evident (even in lobe X). Also, at P35, lobe X had fewer cells than in the wild type (*p* = 0.003; Figure 3A). The differences and changes in the linear density of Purkinje cells could be due to either the number of Purkinje cells, the length of their layer, or both. Consequently, we compared the length of the Purkinje cell layer of lobe X in wild-type and PCD mice at different ages. However, as no differences were found among the different ages or genotypes at each age (*p* > 0.05 in all cases; Supp. Figure S2), the effect of length variation can be discarded. Thus, the aforementioned results regarding density could only be influenced by changes in the number of Purkinje cells.

Concerning the density of HSP25-expressing neurons (Figure 3B), a fluctuating pattern in wild-type mice was detected (pWT = 0.007), but with no apparent drastic changes. Conversely, this density slightly decreased from P15 to P20 in PCD mice and then its expression quadrupled at P25. The density slightly decreased, with no statistical significance, and was maintained until at least P35 (pPCD = 0.009). When the expression of HSP25 was compared between groups, both genotypes at P15 and P20 presented similar densities (*p* > 0.05 in both cases; Figure 3B). However, at P25 and P30, the density of HSP25-expressing neurons was higher in PCD than in wild-type mice (pP25 = 0.008 and pP30 = 0.03, where *p*## is the *p*-value of comparisons between genotypes at each corresponding age; Figure 3B). Finally, at P35, no significant differences were found, most probably due to neuronal loss in the PCD mice.

We also analyzed the percentage of Purkinje cells expressing HSP25 (Figure 3C). For the wild-type mice, different percentages of expression were detected at two separate stages over time (*p* = 0.007; Figure 3C): a basal stage ranging from P15 to P30 and another stage at P35 when the percentage of expression increased. Conversely, these fluctuations differed in PCD mice (*p* = 0.009). In this case, two different stages were identified as follows: one which ranged from P15 to P20 and the second one in which expression increased from P25 to P35 (Figure 3C). In this second stage, at P35, the percentage of HSP25 expression was even greater than 100%. That is, there would be more cells expressing HSP25 than calbindin. This paradox is due to the idea that, at this final point of neurodegeneration, some Purkinje cells stop expressing certain genes and proteins [11], like calbindin, even though they are positive for HSP25 (see Discussion). Upon comparing the percentages between the two groups of animals at different ages, no differences were found at P15 and P20, but at P25, P30, and P35, PCD mice showed higher percentages of Purkinje cells expressing HSP25 than the wild-type mice (pP25 = 0.008, pP30 = 0.003, and pP35 = 0.002; Figure 3C).

### 2.2. Phosphorylation of HSP25 Is More Evident in PCD Mice

**Figure 4:**
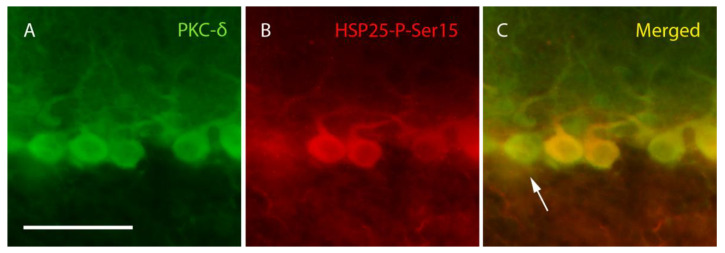
**PKC-δ and HSP25-P-Ser15 labeling.** Images corresponding to lobe X of a PCD mouse at P25 showing four complete Purkinje cells labeled with PKC-δ (green, (**A**,**C**)). Three of these neurons colocalize with HSP25-P-Ser15 (red, (**B**,**C**)), but some exceptions can be also observed (arrow). *n* = 5 for this experiment. Scale bar 50 µm.

Once calbindin and HSP25 expression in Purkinje cells of lobe X was measured, the expression of the active anti-apoptotic isoform HSP25-P-Ser15 was analyzed, as well as the main enzyme responsible for its phosphorylation in the cerebellum, PKC-δ. The labeling for both proteins was limited to the soma of the Purkinje cells and the start of the dendritic tree (Figure 4). However, such labeling was not specific to Purkinje cells only (see below), but as these neurons presented a characteristic morphology and situation, it was easy to distinguish them from other cell types. The number of Purkinje cells showing detectable HSP25-P-Ser15 immunolabeling was markedly lower than those labeled for total HSP25, and both were less abundant than calbindin-positive cells. In fact, the expression of HSP25-P-Ser15 was again almost exclusively relegated to lobe X, except for some sporadic cells in lobe VI. In contrast, PKC-δ expression was found in all cerebellar lobes (Supp. Figure S3), although with qualitative differences among them. The highest expression appeared in lobe X but was lower in the adjacent lobe IX. In the rostral-most areas, no more than five Purkinje cells were detected per lobe, except for lobe VI, where a slightly higher expression of PKC-δ than in its contiguous lobes was detected. Due to this difference in the expression of both proteins, it was easy to find PKC-δ-positive Purkinje cells and HSP25-P-Ser15-negative cells but not vice versa (see also below; Figure 4).

As described in Section 4, the sequential immunohistochemistry used to label PKC-δ and HSP25-P-Ser15 could increase the risk of nonspecific cross-labeling. As PKC-δ labeling was performed after the labeling of HSP25-P-Ser15, if any unspecific binding existed, it would be that the secondary antibody used to detect PKC-δ would adhere to and nonspecifically label the HSP25-P-Ser15 primary antibody. To verify that this was not the case, we looked for HSP25-P-Ser15 labeling that did not co-localize with PKC-δ labeling. This specific staining was difficult to find as the presence of HSP25-P-Ser15 also largely depends on the expression of PKC-δ. Nonetheless, single HSP25-P-Ser15 labeling (without PKC-δ) was detected (Supp. Figure S4), which validated the use of this technique.

**Figure 5:**
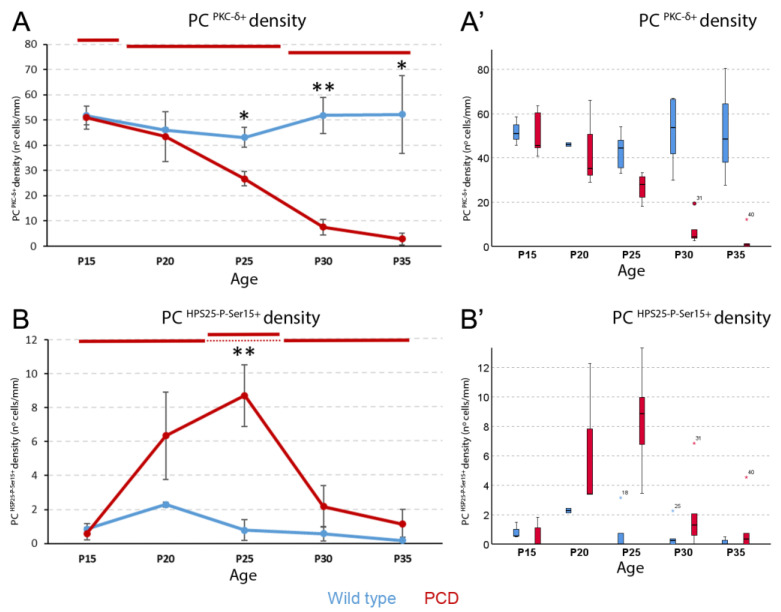
**Analysis of HSP25 phosphorylation in Purkinje cells.** (**A**), density of PKC-δ-positive Purkinje cells. No temporal changes were observed in wild-type animals, whereas in PCD mice, three descending stages are distinguished at P15, at P20-P25, and at P30-P35. Comparison between genotypes showed that at P25, P30, and P35 PCD mice have a lower expression of this protein. (**B**), density of HSP25-P-Ser15-positive Purkinje cells. As before, no changes were observed over time in wild-type animals. In contrast, a peak of expression at P25 is observed in PCD mice. In (**A**,**B**), data are expressed as the mean ± standard error of the mean for easing the comprehension of results. When the Kruskal–Wallis test showed significant differences among ages within each experimental group, a *post hoc* test was applied to define statistically similar groups (subset of values). To identify these subsets and avoid blurring of data and symbols, upper horizontal lines were drawn in each graph for each experimental group (blue, wild type; red, PCD). Note that lines at the same height imply comparable values. (**A’**,**B’**), box plot representations of the same data in A and B, for a better visualization of their distribution, as they were analyzed with non-parametric tests; note that no marks of signification were represented here to avoid blurring the data. PC, Purkinje cell. *n* = 5 for each genotype and age, except for PCD at P30 (*n* = 7) and PCD at P35 (*n* = 8). * *p* < 0.05 and ** *p* < 0.01 for comparisons between wild-type and PCD mice at the corresponding age.

PKC-δ expression did not vary over time in the control animals (*p* > 0.05; Figure 5A; Supp. Figure S3), but progressively decreased in PCD animals (*p* = 0.001; Figure 5A). Regarding the latter, we detected a first stage at P15 with the maximum density of PKC-δ-positive Purkinje neurons (similar to wild type animals), a second stage at P20 and P25 when PKC-δ expression decreases, and a final stage at P30 and P35 when only a few cells were found to be expressing PKC-δ. When comparing both experimental groups, the density of Purkinje cells expressing PKC-δ was significantly lower in PCD than in wild-type mice at P25 (*p* = 0.016), P30 (*p* = 0.008), and P35 (*p* = 0.036).

Concerning the expression of HSP25-P-Ser15, it also remained unchanged over time in wild-type mice (Figure 5B; Supp. Figure S4), with values no greater than two cells per millimeter. In contrast, in PCD mice, the cell density of HSP25-P-Ser15 changed over time (*p* = 0.01; Figure 5B), where the expression started off being similar to that of wild-type mice, then increased until P25, and then returned to its original levels, probably due to Purkinje cell death (see Discussion). P25 is the age when HSP25-P-Ser15 expression is the highest in PCD mice, which is the only age that significantly differs from that of wild-type animals (*p* = 0.008; Figure 5B). Although the focus of this work was the analysis of lobe X, the quantification of PKC-δ and HSP25-P-Ser15-positive Purkinje cells was performed in other lobes. In any case, no evident differences between genotypes were found at critical ages (Supp. Figure S4).

**Figure 6:**
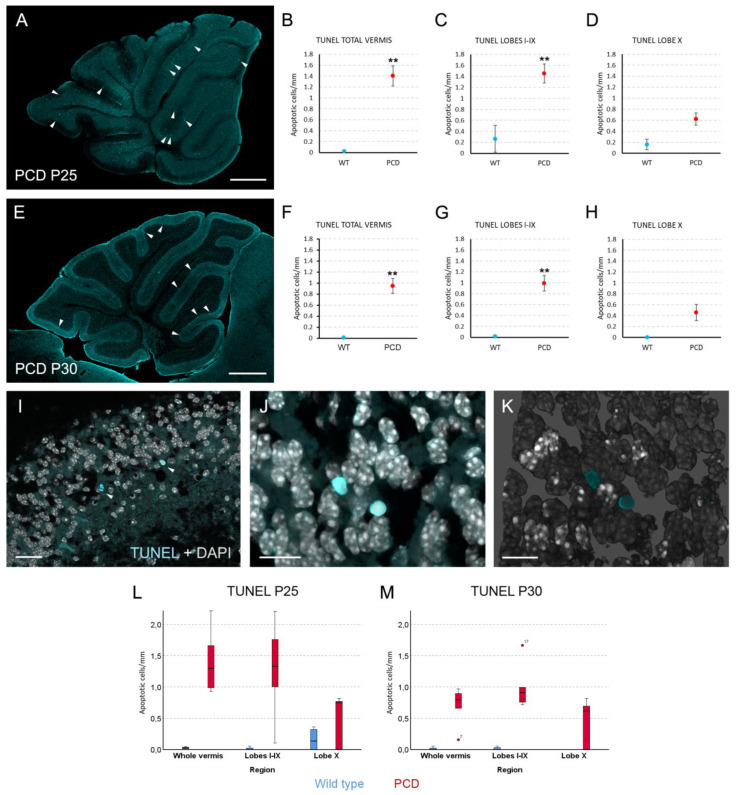
**Analysis of cell death.** (**A**,**E**), sagittal sections of the vermis of PCD mice at P25 (**A**) and P30 (**E**) in which apoptotic cells are labeled with TUNEL (cyan); arrowheads point to examples of apoptotic nuclei in the Purkinje cell layer. (**B**–**D**), charts showing the density of apoptotic cells in the Purkinje cell layer of wild-type and PCD mice at P25 in the whole vermis (**B**), in the lobes I–IX (**C**), and in lobe X (**D**). (**F**–**H**), charts showing the density of apoptotic cells in the Purkinje cell layer of wild-type and PCD mice at P30 in the whole vermis (**F**), in the lobes I-IX (**G**) and in lobe X (**H**). Note that lobe X does not present differences between genotypes due to its neuroprotection. (**I**–**K**), magnified images of apoptotic nuclei in lobes I–IX: focal plane with two apoptotic events in the Purkinje cell layer (arrowheads; **I**), maximum projection of a Z-stack confocal images showing other two TUNEL-positive nuclei (**J**), and 3D reconstruction of the cerebellar cortex with the previous mentioned nuclei (**K**). (**L**,**M**), box plot representations of the same data in (**B**–**D**,**F**–**H**), for a better visualization of their distribution, as they were analyzed with non-parametric tests; note that no marks of signification were represented here to avoid blurring the data. *n* = 5 for each genotype and age, except for PCD at P30 (*n* = 7). Scale bars 400 µm for (**A**,**E**), 20 µm for (**I**), and 10 µm for (**J**,**K**). ** *p* < 0.01.

Finally, we wanted to confirm our results by two additional experiments. First, even when the reduction in Purkinje cells was evident in PCD mice with clear differences between lobes I-IX and lobe X, we wanted to analyze neuronal death directly. To this end, a terminal deoxynucleotidyl transferase-mediated dUTP nick end labeling (TUNEL) assay was performed. We failed to adequately combine immunohistochemistry against calbindin and TUNEL, as very scarce elements presented both labelings. This result is due to the extremely aggressive and rapid death of Purkinje cells, as well as the fact that these neurons stop expressing many proteins—including calbindin—before dying. Therefore, we analyzed the density of TUNEL-positive nuclei in the Purkinje cell layer as an estimator of such neuronal death. TUNEL staining was present in the whole cerebellar parenchyma (Figure 6A,E), also being more abundant in PCD than in wild-type mice. When comparing the density of TUNEL events in the Purkinje cell layer, statistically significant differences amongst genotypes appeared for the whole vermis and the lobes I-IX at both P25 (Figure 6B,C; *p* = 0.01 for both comparisons) and P30 (Figure 6F,G; *p* = 0.01 for both comparisons). However, lobe X did not present differences in cell death between experimental groups at any of the analyzed ages (Figure 6D,H; *p* = 0.171 for P25 and *p* = 0.114 for P30). These results support the neuroresistance of lobe X in comparison with the rest of vermis. Moreover, the density of TUNEL-positive elements was clearly higher in lobes I-IX of PCD mice at P25 compared to P30, which supports the ongoing neuronal death of this region. On the contrary, lobe X did not present noticeable differences in TUNEL staining between ages, presumably due to its neuroprotection.

**Figure 7:**
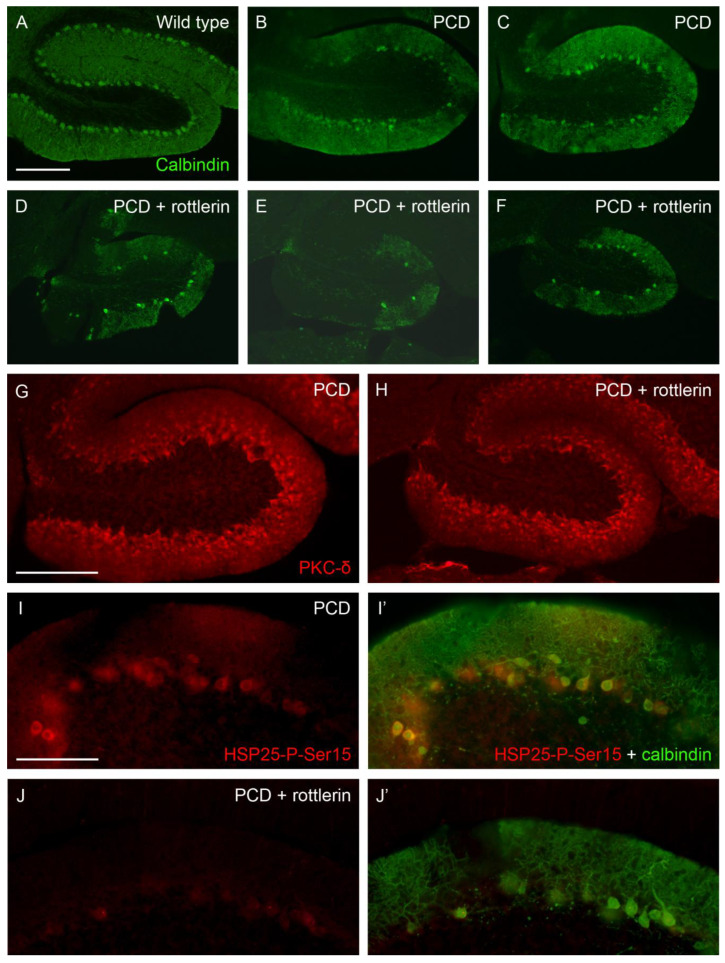
**Effect of rottlerin (PKCδ inhibitor).** (**A**–**F**), sagittal sections of lobe X of wild-type (**A**), untreated PCD (**B**,**C**), and PCD mice administered with rottlerin (**D**–**F**) at P30, in which Purkinje cells are labeled with calbindin (green); note that the Purkinje cell layer is preserved in standard mutant mice, but rottlerin prevented this protection. (**G**,**H**), sagittal sections of lobe X in mutant mice; the staining of PKC-δ (red) is similar in PCD animals independently the administration of rottlerin. (**I**–**J**), HSP25-P-Ser15 labeling (red) in lobe X of PCD mice is dramatically reduced by rottlerin administration. (**I’**–**J’**) calbindin staining (green) is overlaid to the HSP25-P-Ser15 one; note that in untreated PCD mice virtually all Purkinje cells at P30 express the active form of HSP25 (**I’**), whereas scarce neurons do that in rottlerin-administered mutants, and such expression is much weaker (**J’**). *n* = 2 for each genotype in this experiment. Scale bar 200 µm for (**A**–**H**) and 100 µm for (**I**–**J’**).

Our second round of confirmative experiments implied the use of rottlerin, an inhibitor of PKC-δ, which prevented the phosphorylation of HSP25 and, thus, its active form. We injected this compound in PCD mice to demonstrate the neuroprotective effect of HSP25-P-Ser15 in lobe X. We also administered rottlerin to wild-type animals bearing in mind any putative toxic effect of the substance. At P30, lobe X of both wild-type and untreated PCD mice presented a well-preserved Purkinje cell layer (Figure 7A–C), whereas PCD animals administered with rottlerin showed a clear alteration in this structure, with an evident reduction in the number of Purkinje cells and noticeable gaps of their dendritic arborization in the molecular layer (Figure 7D–F, several examples). Then, the expression of PKC-δ was compared in both untreated and rottlerin-administered PCD mice. Our results did not show evident changes in the lobe X of mutants, thus discarding any influence of rottlerin on the distribution of the kinase (Figure 7G,H). On the contrary, our results demonstrated the effect of rottlerin on the inhibition of PKC-δ function, since scarce cells appeared positive for HSP25-P-Ser15 in lobe X of treated animals, and its labeling was clearly weaker than in untreated mice (Figure 7I–J). Even in the regions of lobe X with more Purkinje cells in rottlerin-treated PCD mice, the staining of HSP25-P-Ser15 was very reduced (Figure 7I’–J’). Finally, to discard any toxic effect of rottlerin that may have induced additional Purkinje cell death in PCD mice rather than prevent neuroprotection mediated by HSP25-P-Ser15, the vermis of wild-type mice administered with rottlerin was also analyzed. Since any apparent effect was detected in Purkinje cells, which maintained their layering and structure in whole vermis (Supp. Figure S5), those hypothetic secondary effects of rottlerin were excluded.

## 3. Discussion

### 3.1. Differences Between the Purkinje Cell Densities of PCD and Wild-Type Animals Appear Before Neurodegenerative Stages

The resistance of lobe X was verified in PCD mice, although it was not maintained over time and, at P35, a reduction in Purkinje cell density was detected in these animals. It should be kept in mind that neurodegeneration starts in the rest of the cerebellum at P18 [5] and thus is delayed for approximately 15 days in lobe X. Considering the severity of cell death in this mouse model, 15 days of resistance is significant and Purkinje cells in lobe X last approximately twice as long compared with the rest of the cerebellum.

Before their delayed neurodegeneration, the lobe X of PCD mice had the same Purkinje cells as wild-type animals, except at P15, when the lobe X of wild-type animals shows a linear density of Purkinje cells higher than that of PCD. It should be remembered that at P15, neuronal loss has not yet started in the cerebellum [10]. Moreover, at P20, the neuronal densities of both experimental groups become similar due to a reduction in the wild-type mice, and since we have not detected variations in the length of the Purkinje cell layer. Therefore, this premature difference in the number of Purkinje cells should not be due to neuronal death in the PCD mouse. As such, one hypothesis to explain this phenomenon is neuronal pruning: through this process, large groups of axons, dendrites, and synapses, which are initially supernumerary, are eliminated during the development of the central nervous system, while others are reinforced to achieve more mature, efficient, and precise neural circuits [29]. Neuronal pruning has been shown to occur in the cerebellum in several stages [29]: (1) in prenatal development, when several climbing fibers come into contact with the Purkinje cell bodies; (2) from P0 to P11, when some climbing fibers are removed and others are relocated; (3) from P12 to P17, when more connections are removed; and (4) in adulthood, when those connections that have been maintained previously mature.

In terms of cell number, neuronal apoptosis is known to play a crucial role during the development of the nervous system [30]. During development, neurons are overproduced, and those cells failing to usefully innervate a target are eliminated by apoptosis [31]. The decrease in Purkinje cell density observed in wild-type mice from P15 to P20 coincides temporarily with periods of neuronal pruning. Furthermore, at P15, wild-type mice have a higher cell density in lobe X than PCD animals, which did not exhibit these changes. Then, it is possible that the *pcd* mutation also affects neuronal pruning before the onset of Purkinje cell loss and related symptoms.

Two stages have been described in the cerebellum of PCD mice: pre-neurodegenerative at P15–P17 and neurodegenerative at P18 and onward [10]. It is striking that the pre-neurodegenerative stage coincides temporarily with a clear stage of neuronal pruning and with the lowest Purkinje cell density in PCD mice at P15. A characteristic of the pre-neurodegenerative stage is that the main dendrite of Purkinje cells is shorter and narrower than in wild-type mice [10,32], and this fact could affect the synapses of climbing fibers and, subsequently, neuronal pruning. Therefore, a certain number of Purkinje cells might not be properly innervated and, consequently, be eliminated at P15 by apoptosis [31], affecting their density at this age. We have not found data in the literature about neuron pruning in PCD mice or its relationship with the pre-neurodegenerative stage, so it would be interesting to study this process in more depth, but it is beyond the scope of this work.

### 3.2. HSP25 Is a Neuroprotective Factor in Lobe X of the Cerebellum in PCD Mice

Lobe X of the cerebellum is more resistant than the other lobes in numerous animal models of Purkinje cell death. Examples include the Leaner [33], Toppler [34], Robotic [35], Shaker [36], Lurcher [19], and NPC1 models [20]. We have proposed HSP25 as a candidate for such neuroprotection and verified that the lobe X of PCD mice shows increased expression of this protein compared with that of wild-type animals at P25 and P30. This increased expression coincides temporarily with the prolonged survival of Purkinje cells in lobe X, while the rest of the cerebellum in the PCD mouse undergoes dramatic degeneration [5]. Such a coincidence supports the idea that HSP25 may be one of the factors by which this region resists neurodegeneration longer, both in the PCD mouse and in other models of cerebellar degeneration. In this regard, in the NPC1 [3] and Lurcher models [19], an increased expression of HSP25 in lobe X has also been shown, confirming the neuroprotective role of this protein. Moreover, when analyzing the ratio of HSP25-expressing cells to Purkinje cells, we observed that this value is close to 100% in PCD mice from P25 onward. This means that virtually all Purkinje cells in lobe X also express HSP25. If we consider this moment of strong cerebellar neurodegeneration, the only cells that remain alive are those expressing HSP25, and this protein emerges as a remarkable neuroprotective factor, thus supporting our hypothesis. Furthermore, some calbindin-negative HSP25-positive neurons were detected in the Purkinje cell layer. Calcium-binding proteins as calbindin or parvalbumin are widely considered as markers of Purkinje cells [2,10]. However, there are scarce subpopulations of these neurons that result negative for one of these markers. In addition, PCD neurodegeneration is accompanied by gene and protein silencing before neuronal death [11], which includes neuronal markers. Therefore, it is plausible to think that damaged Purkinje cells could stop expressing some genes/proteins, but maintaining the production of putative neuroprotective factors (i.e., HSP25) as we propose here.

There is a constitutive expression of HSP25 defined in a stripe pattern, which is especially abundant in the nodular zone [15,19,37]. In this sense, it seems that HSP25 is a neuroprotective factor that is more abundant in this region, as shown in wild-type mice, and whose expression is increased during a neurodegenerative process. The progression of Purkinje cell death was studied in coronal sections in NPC1 mice, and, at P240, the largest number of surviving Purkinje cells was found in a central band including lobe X [3]. This band also coincides with one of the stripes—the most medial—in which HSP25 is expressed [38]. Interestingly, HSP25 is also expressed, albeit weakly, in the central area of the cerebellum, particularly in lobe VI [20]. In the NPC1 model, this region is slightly resistant to neurodegeneration [20], which also supports the theory that where HSP25 is constitutively expressed, it somehow prevents neurodegeneration in a proportional manner. The expression of HSP25 in lobe VI is less evident than in lobe X and consequently, although there is some resistance in lobe VI, it has been described in far fewer models than lobe X resistance (the PCD mouse is not an example of this).

Here, it is important to note that other additional mechanisms of neuroresistance can overlap with the basal neuroprotection of HSP25. We have recently demonstrated that lobe X is less dependent on the expression of *Ccp1* gene and CCP1 protein, which may confer an additional resistance to their Purkinje neurons in PCD mice [12]. It is possible that in other models of cerebellar damage in which lobe X resistance had been detected, different and non-excluding mechanisms of neuronal survival could be identified. The coexistence of both basal neuroprotection and specific neuroresistance mechanisms in the same structure makes lobe X a privileged cerebellar region, probably by different evolutionary or phylogenetic reasons [4].

### 3.3. PKC-δ Is Constitutively Expressed and HSP25-P-Ser15 Is Upregulated During Neurodegeneration

We have verified that the expression of PKC-δ is constant over time in lobe X of wild-type mice, and its cell density is around 50 cells/mm, similar to calbindin expression. Thus, it appears that PKC-δ expression is almost constitutive in the Purkinje cells of lobe X, as has been observed by other authors [38]. In lobe IX, on the other hand, PKC-δ expression is somewhat lower than in lobe X, being occasional in rostral-most areas, except for lobe VI, where it is increased (see below discussion concerning HSP25-P-Ser15). Therefore, PKC-δ expression is higher in the most resistant lobes, and it would be logical to think that this enzyme has indirect neuroprotective effects (at least when related to HSP25), as has been previously shown [21]. In parallel, the constitutive expression of PKC-δ in lobe X makes sense, as its role is not limited to phosphorylating HSP25, but has multiple functions in the cell [39].

As for the presence of HSP25-P-Ser15, we found that although its expression is quite low in lobe X of PCD mice, it is higher than that of the wild type at P25. We found no expression in the rest of the lobes, except in some sporadic cells in lobe VI of mutant animals. Within lobe X, HSP25-P-Ser15 expression was detected in a small number of Purkinje cells. Unlike total HSP25, its distribution did not show a clear ventral predominance, likely due to the low abundance of this phosphorylated form. This finding supports the idea of a putative mild resistance of lobe VI (see discussion above regarding the presence of the kinase that generates this phosphorylated form in lobe VI). In wild-type mice, we also found almost no labeling, suggesting that the presence of HSP25-P-Ser15 is also consistent with an induction process associated with neurodegeneration in the PCD mouse. It is important to note that the quantification of HSP25-P-Ser15 was based on a relatively small number of positive Purkinje cells, which reflects the low abundance of this phosphorylated isoform under both physiological and pathological conditions rather than a technical limitation. Similar low expression levels have also been reported in other models showing HSP25-P-Ser15 upregulation during neurodegeneration [21]. Although the reduced number of labeled cells may increase variability and limit the statistical power of the analysis, we used non-parametric statistical tests appropriate for small sample sizes, and the observed temporal pattern was consistent across animals and in line with our working hypothesis. Indeed, we found a similar temporal expression pattern in both the phosphorylated and non-phosphorylated forms of the protein: initially, at P15, there is a relatively low expression that begins to increase to a maximum at P25, just before the onset of lobe X neurodegeneration. From this age onward, the density of Purkinje cells expressing HSP25-P-Ser15 decreases, probably due to their death. As observed with HSP25, the presence of HSP25-P-Ser15 also may reflect an induction process during neurodegeneration, although HSP25 expression arises from a basal/constitutive presence, unlike the case of HSP25-P-Ser15, which is scarce without any stimulus. Chung et al. in 2016 [21] verified the presence of HSP25-P-Ser15 and PKC-δ in the nodular and posterior regions of the NPC1 mouse, corresponding to those lobes that showed the highest resistance in this model. In addition, they induced or inhibited the expression of PKC-δ and HSP25 in this animal, showing that the neurodegenerative and behavioral effects were ameliorated or aggravated, respectively. Finally, they demonstrated how the existence of PKC-δ increased the presence of HSP25-P-Ser15, which also had potent neuroprotective effects [21]. These results confirmed the neuroprotective effect of the phosphorylated version of HSP25 in the mouse cerebellum. Thus, we can say that the resistance of lobe X of the cerebellum is also related to HSP25-P-Ser15, and the protein responsible for phosphorylating HSP25 in our model may be PKC-δ.

When comparing the results of HSP25 with those for HSP25-P-Ser15, they show a large difference. Considering P25 as a reference, the time of maximum expression of both protein forms in PCD mice, the number of HSP25-positive neurons is around 40 cells/mm while the number of HSP25-P-Ser15-positive neurons is about 8 cells/mm. The explanation for this difference is simple: the presence of phosphorylated HSP25 necessarily implies the prior existence of unphosphorylated HSP25, in addition to PKC-δ, which we have found not to be a limiting factor in this case. Likewise, our results demonstrate that the production of both forms of this protein can be induced, to maintain neuronal homeostasis and confer neuroprotection: HSP25 as a chaperone [22] and HSP25-P-Ser15 as an anti-apoptotic factor [28]. In fact, one of the first detrimental effects suffered by Purkinje cells in PCD mice is the destabilization of the cytoskeleton [9,10], which makes sense for the initial increase in the expression of a chaperone that stabilizes other proteins [14,15,22]. In any case, it cannot be discarded as a neuroprotective role of HSP25 itself, at it has been recently demonstrated in the cerebellum [40]. The ways for exerting neuroprotection (apart from its anti-apoptotic active form) could be variated, and may be related with regulating oxidative stress and neuroinflammation [41]. Then, when the neurodegenerative process progresses and neuronal death begins, molecules that specifically counteract apoptosis, such as HSP25-P-Ser15, would be needed. Therefore, what could happen in lobe X of PCD mice is that before neurodegeneration, when cell damage before Purkinje cell death begins, neuroprotective factors start to be expressed in such a way that HSP25 expression increases rapidly. Indeed, one characteristic of the DNA encoding HSPs is that it lacks introns [42]. Thus, both HSP mRNA and protein can be produced rapidly upon a stressful stimulus because the mRNA does not require splicing [42]. Apart from the aforementioned models of cerebellar damage, there are recent data demonstrating an increase in HSP25 against different types of neuronal damage, not only in the cerebellum [40], but also in other regions of the central nervous system [43]. Indeed, the prevention of the expression of HSP25, precisely fosters the cell damage [44]. However, the phosphorylation of this protein is a process that must necessarily begin after the presence of HSP25. Therefore, when HSP25-P-Ser15 expression is finally increased, it may be too late and Purkinje cell death has already begun, leading to a decrease in the number of HSP25-P-Ser15-positive Purkinje cells. In addition, one of the effects of the *pcd* mutation in Purkinje cells is an accumulation of DNA damage, leading to a compaction of chromatin that prevents it from being read and inhibits the expression of certain proteins [45]. Thus, even if they benefit from neuroprotection, Purkinje cells in lobe X may undergo gene silencing that could eventually affect the expression of HSP25 or HSP25-P-Ser15 and aggravate the symptomatology of this cell type.

Finally, our last confirmative experiments validated our study. Consistently with previous reports, we observed that Purkinje cells expressing HSP25 tend to persist longer (lobe X), whereas those lacking HSP25 are more vulnerable to degeneration (lobes I–IX). The use of direct apoptotic markers, such as TUNEL, confirmed this relationship. Moreover, our observations strongly support an association between HSP25/HSP25-P-Ser15 expression and the resistance of lobe X Purkinje cells. The administration of rottlerin, a PKC-δ inhibitor, dramatically reduced the extent of HSP25-P-Ser15, the active form of HSP25. Such inhibition clearly reduced the neuroprotection of lobe X, as it suffered an evident Purkinje cell loss in PCD mice treated with rottlerin. These results clearly establish a direct causality amongst HSP25/HSP25-P-Ser15 expression and the neuroprotection of lobe X. Similar approaches have been successfully applied in other models, such as the NPC1 mouse [21]. It is necessary to note that despite the additional Purkinje cell loss caused by rottlerin administration in PCD mice, the lobe X of these animals still presented some Purkinje cells, with a density higher than in the rest of the vermis. This residual survival may reflect an additional lower vulnerability of lobe X to the lack of CCP1, as we have recently demonstrated [12]. In any case, an incomplete effect of rottlerin cannot be discarded.

### 3.4. Limitations

We have validated the function of HSP25 (and its active form, HSP25-P-Ser15) as neuroprotective molecules induced by neurodegenerative processes. However, it is necessary to mention some limitations of our study.

First, our study is focused on lobe X of the cerebellum in standard PCD mice. It would be interesting to extend this study to other lobes partially protected by HSP25 (i.e., lobe VI), if not in this specific model, in others analogous: other *pcd* mutations with milder effects, inducible mutants, etc.

Second, the quantification of apoptotic nuclei in the Purkinje cell layer was not combined with immunohistochemistry against calbindin or other Purkinje cell markers by technical troubles (see above). Of course, we cannot discard a minority quantification of other apoptotic cells in this layer, but this number is negligible in comparison with the main Purkinje cell death rate. In addition, the quantification in the wild-type group should compensate any bias.

Third, the results comprising the use of rottlerin were qualitative, as their related experiments were confirmative. Fortunately, such results were solid enough to corroborate a cause–consequence relationship between HSP25 and neuroprotection.

## 4. Materials and Methods

### 4.1. Mice and Genotyping

PCD and wild-type mice of the C57/DBA strain were used in this study. All animals were established in a colony at the Animal Experimentation Service of the University of Salamanca. To obtain PCD mice, heterozygous animals (+/*pcd*) were crossed because male PCD mice are sterile and females are poor breeders. Since wild-type and heterozygous mice are phenotypically indistinguishable, and PCD animals were used before the onset of their symptoms (see later), each mouse was genotyped. For this purpose, DNA was extracted from a tissue sample and PCR was performed to amplify the microsatellite regions D13Mit250 and D13Mit283 linked to the gene region. The specific primers employed were 5′-ACACTCATTTCCATGCACGA-3′ (forward) and 5′-AGGTCCTCAAATCTCACAAGTAGG-3′ (reverse) for D13Mit250 and 5′-GGAAGCAGTCTCCTGCCTC-3′ (forward) and 5′-GAGAGGTGGCACATGAGGTT-3′ (reverse) for D13Mit283. Both PCD model as well as primers for its genotyping have been extensively validated [10,11,12,32,41].

The mice were kept, handled, and sacrificed following the requirements of the directive of the Council of the European Communities (2010/63/EU) and Spanish Legislation (RD118/2021) in force for the use and care of laboratory animals. The Bioethics Committee of the University of Salamanca approved the procedures carried out (reference number 291 and 613).

### 4.2. Rottlerin Administration

Two additional mice of each genotype were administered with rottlerin, an inhibitor of PKC-δ [46]. Rottlerin was solved in dimethyl sulfoxide at a concentration of 10 µg/µL and then diluted in phosphate buffer saline (PBS) 1:10 (*v*/*v*). Animals were intraperitoneally administered with this solution at a rottlerin concentration of 8 µg/g of body weight [46] at P24 and P27, that is to say, in the peak of HSP25-P-Ser15 activity in PCD mice.

### 4.3. Tissue Collection

For this work, 55 mice were divided into 10 groups depending on their genotype and age for standard experiments, plus 2 additional mice of each genotype for confirmative experiments using rottlerin (see above; Table 1). The animals were deeply anesthetized, and once they did not have any reflexes or show further signs of consciousness, they were sacrificed by trans-cardiac perfusion with Somogyi’s fixative without glutaraldehyde. After 15 min of perfusion, the cerebellum was removed and immersed in the same fixative for 2 h to post-fix the tissue.

Samples were washed with 0.1 M phosphate buffer (PB) and cryoprotected using a 30% (*w*/*v*) sucrose solution in PB. Once the tissue had sunk to the bottom of the vial, the tissue blocks were sectioned in sagittal sections of 30 µm thickness using a freezing sliding microtome (Jung SM 2000, Leica Microsystems, Wetzlar, Germany). For the immunohistochemical experiments, the sections corresponding to the cerebellar vermis were selected, allowing all cerebellar lobes to be observed in full. Furthermore, the distribution of the expression of HSP25 was confirmed to be present in 3 sagittal bands in coronal slices of one animal for each genotype: one of these being in the central-most area of the vermis [37].

### 4.4. Immunohistochemistry

All the antibodies used in this work and their dilutions are listed in Table 2. After a first washing with PBS (3 × 10 min), sections were incubated for 72 h at 4 °C in primary antiserum comprising Triton X-100 0.2% (*v*/*v*), goat serum 5% (*v*/*v*), the antibodies against calbindin (for labeling Purkinje cells) and HSP25, and with PBS as the diluent. After this first incubation, the sections were washed again with PBS (3 × 10 min) and incubated at room temperature for 90 min in the secondary antiserum comprising the corresponding secondary antibodies bound to Cy2 and Cy3 fluorochromes diluted in PBS. In the last 10 min of incubation, 4′,6-diamidino-2-phenylindole (DAPI; 1:10,000; *v*/*v*) was added to counterstain the cell nuclei. Samples were mounted and covered on a slide with an anti-fade mounting medium.

The analysis of PKC-δ and the phosphorylated version of HSP25 was performed using sequential immunohistochemistry because all available commercial antibodies were made in rabbits. First, the slides were incubated with the primary antiserum containing α-HSP25-P-Ser15 antibody and then incubated with the corresponding secondary antibody to saturation (see below). Once HSP25-P-Ser15 was labeled, a second round of incubations was carried out using the antibody against PKC-δ followed by its corresponding secondary antibody. PBS washes and the first incubation with the primary antibody were performed as described above; the antiserum contained 0.2% (*v*/*v*) Triton X-100, 5% (*v*/*v*) goat serum, and the antibody against HSP25-P-Ser15. After 72 h at 4 °C, the tissue was incubated with the Cy3 secondary anti-rabbit IgG antibody at 1:50 (*v*/*v*) in PBS for 5 h. The concentration of the secondary antibody and the incubation time were notably increased to saturate the epitopes of the primary antibody to prevent the other secondary antibody from binding to the anti-HSP25-P-Ser15 antibody in the subsequent incubation. After completing the labeling for HPS25-P, the sections were washed with PBS (6 × 10 min) and incubated for 72 h at 4 °C with the other primary antiserum containing 5% (*v*/*v*) goat serum and the antibody against PKC-δ in PBS. Finally, the slides were washed with PBS (3 × 10 min) and incubated with the rabbit α-IgG antibody with Cy2 at 1:500 (*v*/*v*) for 1 h; in the last 10 min, DAPI was added at a final dilution of 1:10,000 (*v*/*v*). The protocol was completed by mounting and covering the sections. Specific controls for this unusual procedure were performed to verify the specificity of labeling. In addition, the results of this work also allowed the possibility of cross-labeling to be discarded (see later).

Neuronal death was analyzed by labeling apoptotic cells with TUNEL technique. Tissue slices were washed with PBS (3 × 10 min) and permeabilized with 0.2% (*v*/*v*) Triton X-100 and 0.1% (*p*/*v*) sodium citrate in HELIX water for 15 min. Then, they were washed with PBS and incubated with TUNEL buffer containing 30 mM Tris-HCl, 140 mM sodium cacodylate, 1 mM CoCl_2_ and 0.3% Triton X-100 (*v*/*v*) for 30 min. After that, the slices were incubated in a medium with terminal transferase (800 U/mL; Roche Diagnostics, Mannheim, Germany) and biotinylated dUTP (1 µM; Roche Diagnostics) in TUNEL buffer for 2 h. The reaction was stopped by adding saline sodium citrate buffer (0.15 M sodium chloride and 0.015 M sodium citrate). Lastly, the slices were rinsed with PBS and the staining was revealed with a medium containing Cy2-conjugated streptavidin (1:200; Jackson) in PBS. DAPI staining and slide mounting was performed as described above. The counting of TUNEL-positive cells was restricted to the Purkinje cell layer to determine the density of apoptotic cells. It was also performed in the whole vermis and separately in lobes I-IX and X, in both wild-type and PCD mice at P25 and P30.

Samples were visualized under an epifluorescence microscope (Olympus Provis AX70, Tokyo, Japan). Images were taken using an attached camera (Olympus DP70) and quality was enhanced using Adobe Photoshop Creative Cloud 2015 (Adobe Systems Software, Dublin, Ireland).

### 4.5. Quantification and Statistics

All of the measurements performed refer to lobe X and not to the cerebellar cortex for the following two reasons: (1) lobe X is the tissue of interest in this study as it is the only neuroresistant region, and (2) neurodegeneration in PCD mice progresses so rapidly that at some of the ages studied, no Purkinje cells exist in lobes I-IX, so counting them is useless.

The linear densities of the different markers were calculated by referring the number of positive elements to the length of the Purkinje cell layer in each sagittal section analyzed. The percentage of HSP25-positive cells in the calbindin-positive elements (i.e., Purkinje cells) was also calculated. The data obtained were represented as the mean cell density (cells/mm) or as the mean percentage ± the standard error of the mean, for a more simplistic, better comprehension. In addition, box plots are also provided to offer a more accurate distribution of data. The values corresponding to PCD and wild-type mice were compared at different ages using the Mann–Whitney’s U test. Additionally, comparisons among ages for each experimental group were also performed using the Kruskal–Wallis test to look for possible differences in temporary fluctuations between phenotypes. When significant differences were detected (*p* < 0.05), the post hoc analysis of stepwise step-down comparisons was subsequently applied. This post hoc comparison, analog to the Student–Newman–Keuls test, allowed the statistically similar ages to be classified into the most probable homogeneous subsets of data. Such subgroups are based on asymptotic significations, at a level of significance set at 0.05. Non-parametric tests were chosen due to the size of the experimental groups. All statistical analyses were performed using the SPSS Statistics 26 program (IBM SPSS, Armonk, NY, USA).

## 5. Conclusions

Two main conclusions can be drawn regarding the expression of HSP25 and HSP25-P-Ser15: (1) the resistance of lobe X of the PCD mouse may be due to both constitutive and subsequently induced expression of HSP25; and (2) HSP25-P-Ser15 is not constitutive in lobe X, but may be induced by neurodegeneration, thereby contributing to the preservation of Purkinje cells. Analysis of regions naturally resistant to neurodegeneration may reveal new molecules whose induction could benefit neurodegenerative diseases, and stimulation or administration of such endogenous central nervous system molecules might offer an alternative approach to combat neuronal loss with minimal side effects.

## Figures and Tables

**Table 1:** The mice used in this study sorted by genotype and age group.

	P15	P20	P25	P30	P35
PCD	5	5	5	7	8
Wild type	5	5	5	5	5
PCD + rottlerin				2	
Wild type + rottlerin				2	

**Table 2:** Relevant information about primary and secondary antibodies used in this work.

	Antibody	Dilution	Animal	Producer	Ref./Code
Primary antibodies	Calbindin	1:2000	Mouse	Swant (Burgdorf, Switzerland)	CB300-200uL
	HSP25	1:1000	Rabbit	Enzo Life Sciences (Farmingdale, NY, USA)	ADI-SPA-801
	PKC-δ	1:2000	Rabbit	Invitrogen (Carlsbad, CA, USA)	MA5-32482
	HSP25-P-Ser15	1:1000	Rabbit	BioWorld Technology (St. Louis Park, MN, USA)	BS4762
Secondary antibodies	Cy2 anti-Mouse	1:500	Goat	Jackson ImmunoResearch (West Grove, PN, USA)	115-225-003
	Cy2 anti-Rabbit	1:500	Goat	Jackson ImmunoResearch	111-225-003
	Cy3 anti-Rabbit	1:500 or 1:50	Goat	Jackson ImmunoResearch	111-165-003

## Data Availability

The datasets generated and analyzed during this study are not publicly available due to regulatory restrictions but are available from the corresponding authors upon reasonable request.

## Supplementary Materials

The following supporting information can be downloaded at https://www.mdpi.com/article/10.3390/ijms27031145/s1.

## Abbreviations

The following abbreviations are used in this manuscript:

CONDCA	childhood-onset neurodegeneration with cerebellar atrophy
HSP	heat shock proteins
P#	postnatal (day)
PB	phosphate buffer
PBS	phosphate buffer saline
PCD	Purkinje Cell Degeneration
PKC-δ	protein kinase C-δ
sHSPs	small HSPs
TUNEL	terminal deoxynucleotidyl transferase mediated dUTP nick end labeling
