# Supplementary material for: HSP25 and HSP25-P-Ser15 Prompt Innate Neuroprotection in Lobe X of the Cerebellum
Source: Int J Mol Sci. 2026 Jan 23;27(3):1145. doi: 10.3390/ijms27031145 (PMC12897430; doi:10.3390/ijms27031145)
Supplement: Supplementary file 1 [file ijms-27-01145-s001.zip › Supplementary figure legends V4.pdf]

Supplementary Figure S1. Vermis of a PCD mouse. Photomicrograph of a sagittal section of the vermis of a PCD mouse at P30. Purkinje cells are labeled with calbindin (green). Note the advanced degeneration throughout the entire cerebellar cortex with only isolated Purkinje cells, except in lobe X (marked with an X), where the Purkinje cell layer remains intact. These calbindin-positive Purkinje cells in lobes I-IX represent transiently preserved neurons during the degenerative process rather than long-term resistant populations; at later stages they largely disappear. Image extracted from Hernández Pérez et al. (*Anatomia*, 2023). Scale bar 1 mm.

Supplementary Figure S2. Extension of the Purkinje cell layer in lobe X. A, mean length of the Purkinje cell layer in lobe X of vermis at different ages in wild-type and PCD mice. No changes over time or differences among genotypes were detected at each time point. **A'**, box plot representations of the same data in A, for a better visualization of their distribution, as they were analyzed with non-parametric tests.

Supplementary Figure S3. Labeling and quantification for PKC-δ and HSP25-P-Ser15. **A-C,** low-magnified images of cerebellar vermis corresponding to a PCD mouse at P25; PKC-δ staining (red) extends for all cerebellar lobes, whereas HSP25-P-Ser15 labeling (green) is much more restricted, especially to lobe X (marked with an X). **D**, table showing the mean values of the density of PKC-δ and HSP25-P-Ser15-positive Purkinje cells (nº cells/mm) in both wild-type and PCD mice at critical ages of cerebellar degeneration.

Supplementary Figure S4. Different labeling for PKC-δ and HSP25-P-Ser15. In these images, corresponding to Purkinje cells of a PCD mouse at P25, 3 possible labeling conditions can be verified: Purkinje cells showing both PKC-δ (green, **A**) and HSP25-P-Ser15 (red, **B**) staining (asterisks, **C**), cells with strong PKC-δ and weak HSP25-P labeling (arrowhead, **C**), and a possible displaced Purkinje cell expressing only HSP25-P (arrow, **C**). Cells like the latter demonstrate that non-specific cross-labeling of antibodies has not occurred. Scale bar 50 µm.

Supplementary Figure S5. Vermis of a wild-type mouse administered with rottlerin. Photomicrograph of a sagittal section of the vermis of a wild-type mouse at P30, after being

injected with rottlerin at P24 and P27. Note the complete layer of Purkinje cells labeled with calbindin (green), which discards any toxic effect of the administered substance. Scale bar 500 μm.
