# Supplementary figures and images for: HSP25 and HSP25-P-Ser15 Prompt Innate Neuroprotection in Lobe X of the Cerebellum
Source: Int J Mol Sci. 2026 Jan 23;27(3):1145. doi: 10.3390/ijms27031145 (PMC12897430; doi:10.3390/ijms27031145)

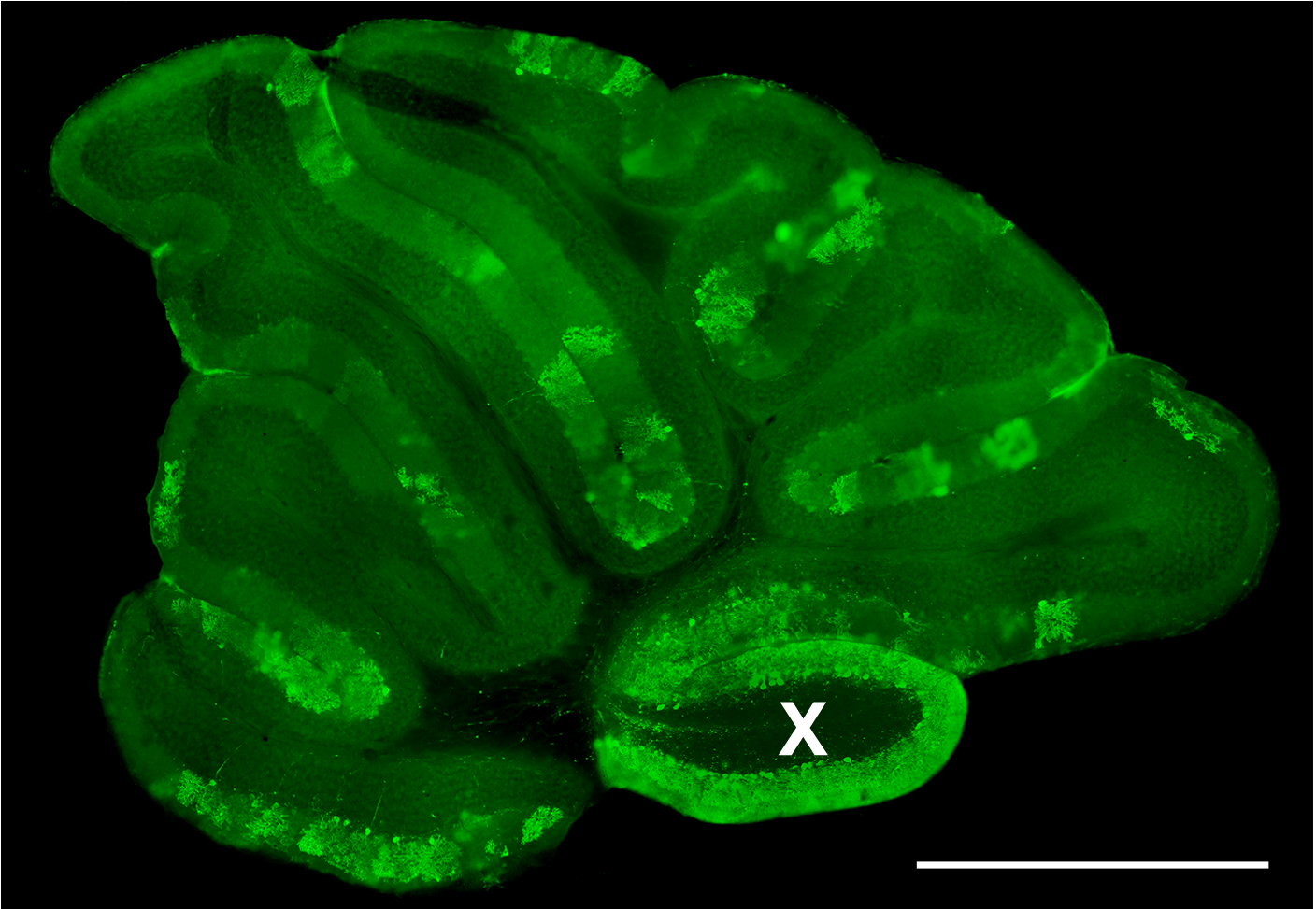

Supplement: Supplementary file 1 [file ijms-27-01145-s001.zip › Supp Figure 1.tif]

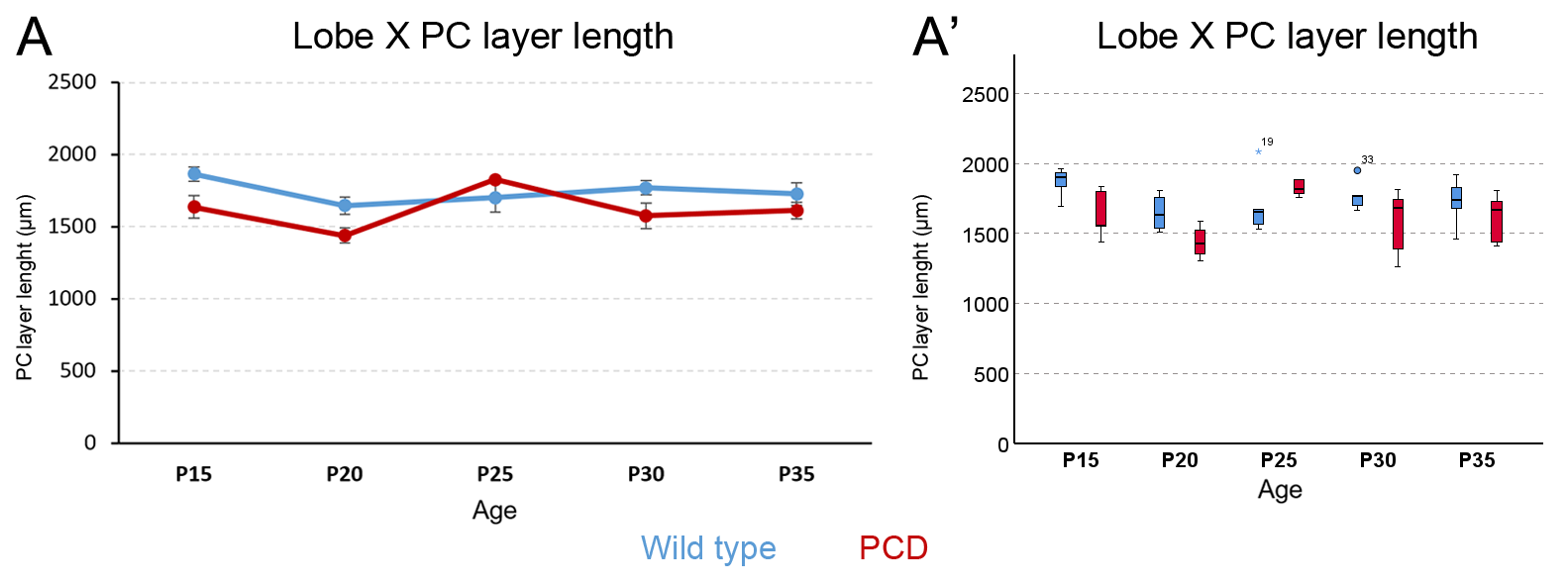

Supplement: Supplementary file 1 [file ijms-27-01145-s001.zip › Supp Figure 2.tif]

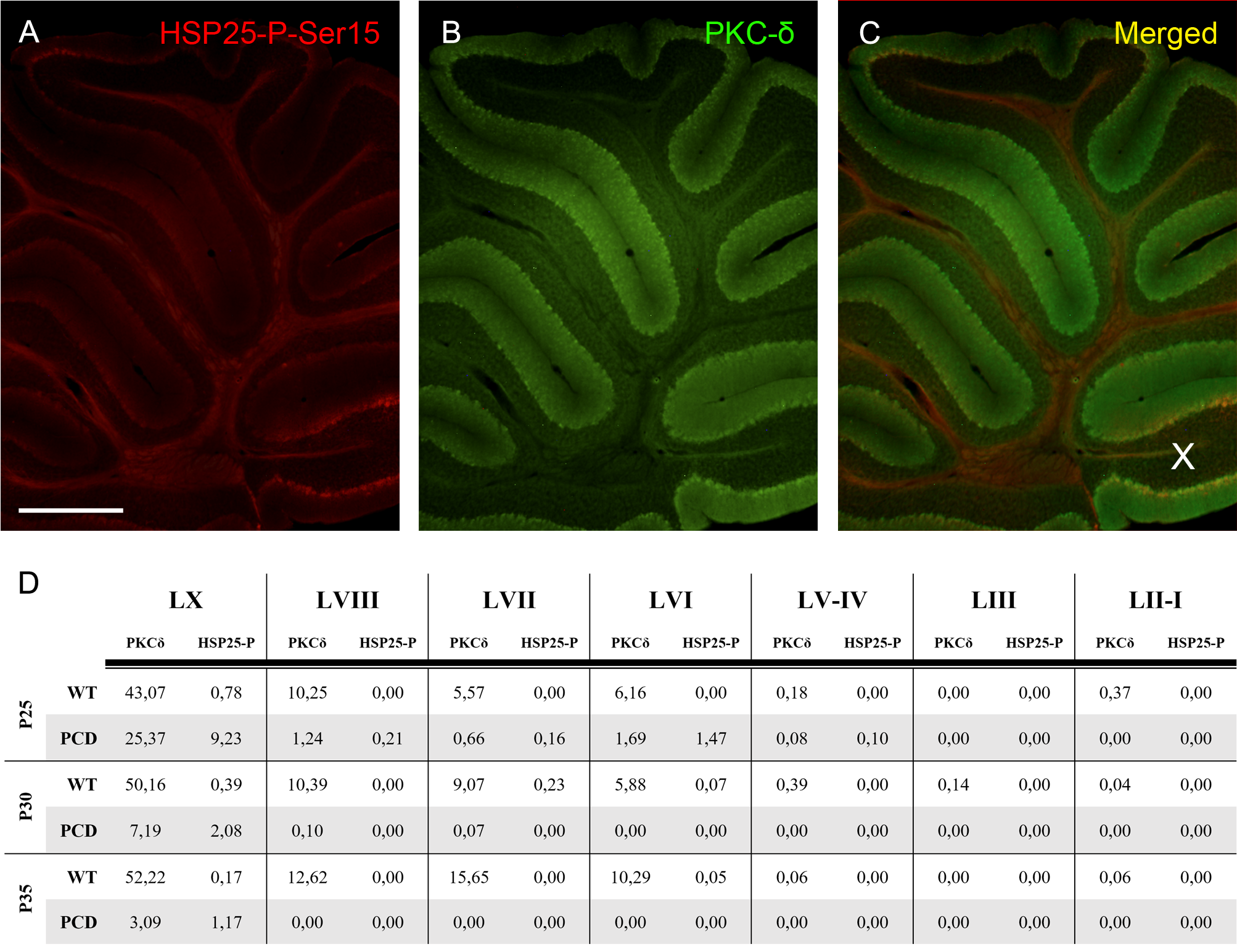

Supplement: Supplementary file 1 [file ijms-27-01145-s001.zip › Supp Figure 3.tif]

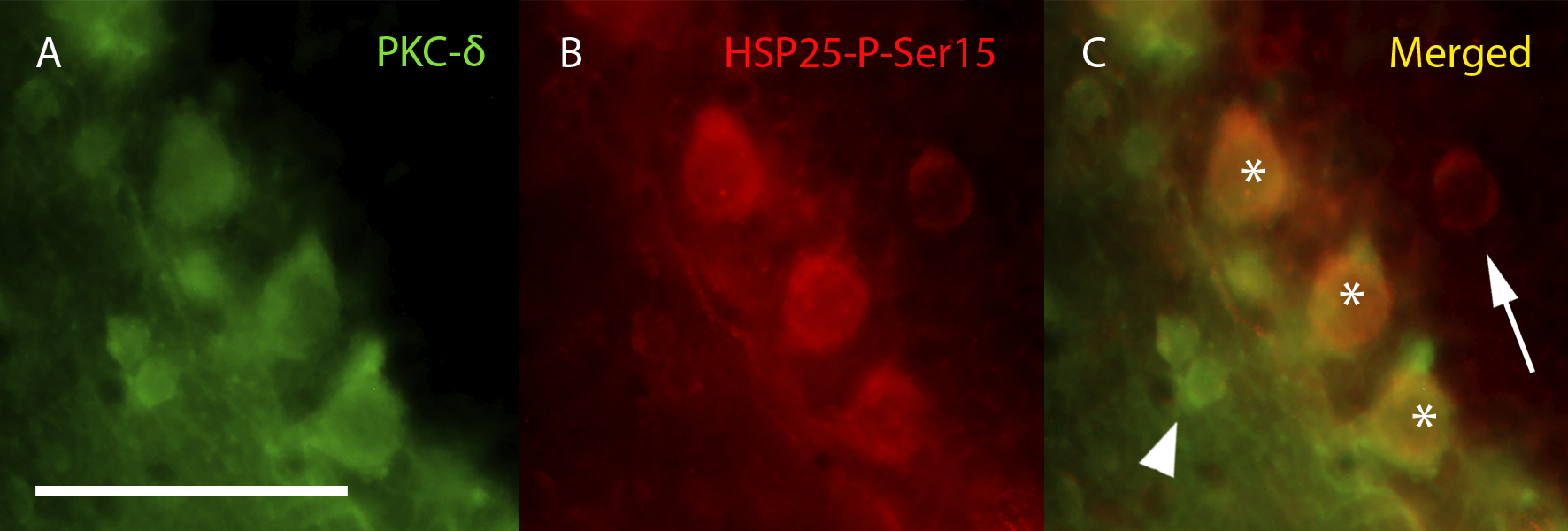

Supplement: Supplementary file 1 [file ijms-27-01145-s001.zip › Supp Figure 4.tif]

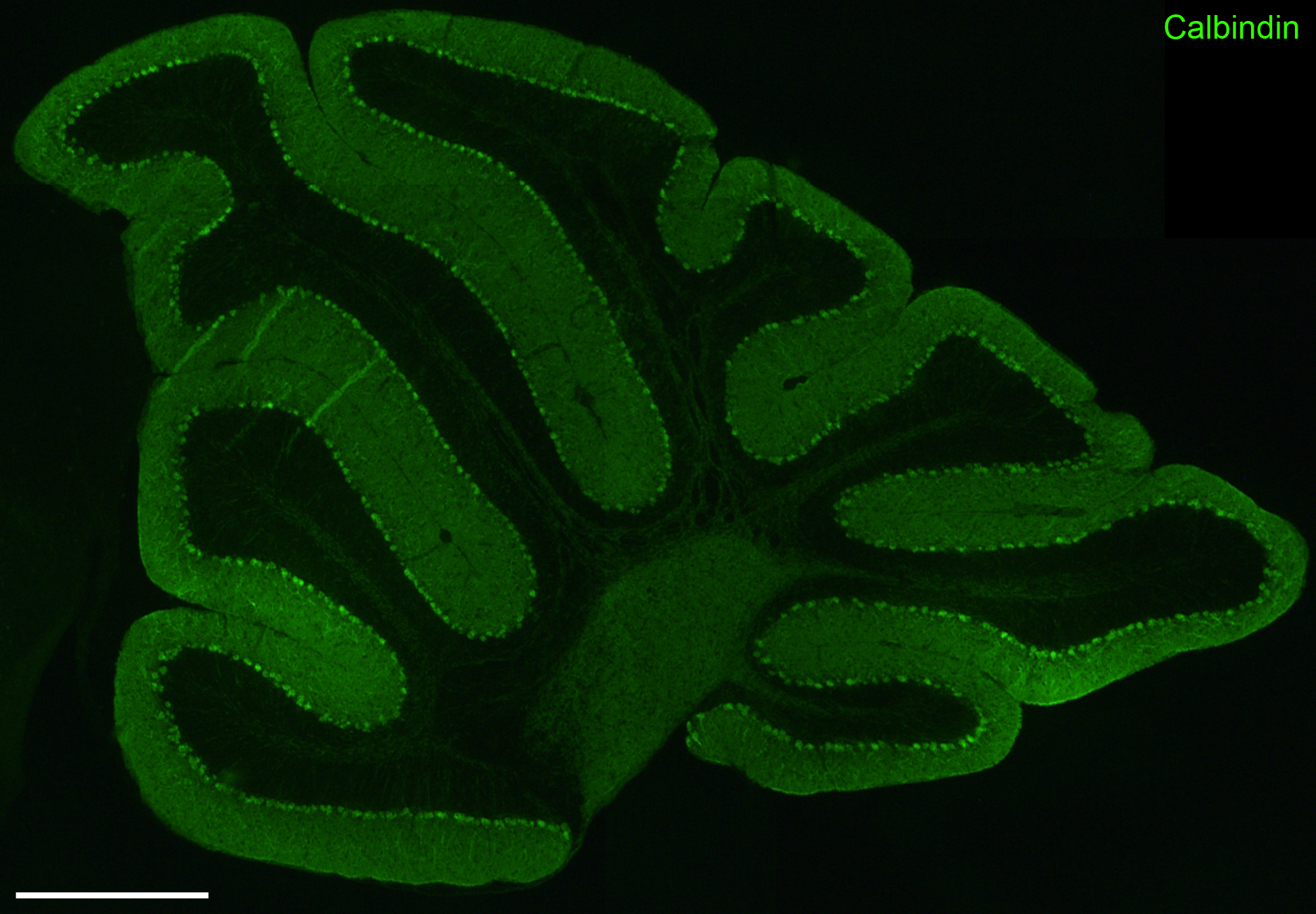

Supplement: Supplementary file 1 [file ijms-27-01145-s001.zip › Supp Figure 5.tif]
